# Evaluating the Effect of High‐Translucent Zirconia Thickness and Substrate Shade on the Final Color of the Restoration

**DOI:** 10.1002/cre2.70091

**Published:** 2025-02-18

**Authors:** Mahnaz Hatami, Elham Jalali, Mohamad Hossein Lotfi Kamran, Alireza Danesh Kazemi, Amirhossein Fathi

**Affiliations:** ^1^ Department of Prosthodontics, School of Dentistry Shahid Sadoughi University of Medical Sciences Yazd Iran; ^2^ Prosthodontist Yazd Iran; ^3^ Department of Operative Dentistry, School of Dentistry Shahid Sadoughi University of Medical Sciences Yazd Iran; ^4^ Dental Prosthodontics Department, Dental Materials Research Center, School of Dentistry Isfahan University of Medical Sciences Isfahan Iran

**Keywords:** color, spectrophotometer, translucency, zirconia

## Abstract

**Objectives:**

The purpose of this study was to evaluate the effect of high‐translucent zirconia thickness and substrate shade on the final color of the restoration.

**Material and Methods:**

A total of 60 high‐translucent monolithic zirconia disks were prepared using a CAD/CAM system. They were placed on composite substrates with A2, A3.5, and C3 colors (*n* = 10). The color differences (Δ*E*) of the ceramic disks on the A3.5 and C3 substrate compared to the control group (A2) were calculated and compared with acceptable thresholds (Δ*E* = 5.5) and perceptible thresholds (Δ*E* = 2/6). A one‐sample *t*‐test and repeated measures ANOVA were used to analyze the data statistically.

**Results:**

The highest Δ*E* values were observed when comparing two zirconia disk thicknesses on the A3.2 substrate without cement (Δ*E* = 5/65). The lowest value of Δ*E* compared to the control group (A2) was related to the disk with 1 mm thickness on the A3.5 substrate (Δ*E* = 2/54), and the highest value was for the disk with 0.6 mm thickness on the C3 substrate (Δ*E* = 4/88).

**Conclusions:**

Using the zirconia disk with a 1 mm thickness on the C3 structure and a disk with a 0.6 mm thickness on the A3.5 structure with the presence of F2 cement can achieve an acceptable color difference (value of 5.5).

## 1 Introduction

The increase in patient expectations of esthetic treatments, biocompatibility and chemical‐resistant composite of ceramic materials has led to the widespread use of all‐ceramic restorations. All‐ceramic crowns are widely used, especially in the anterior teeth, to achieve maximum esthetics. One of the most important advantages of these materials is semi‐translucency, which allows light to pass through the crown (Naert et al. 2005). Conventional zirconia has less translucency (more opacities) than other all‐ceramic systems. So, a layer of porcelain can be attached to the zirconia surface or pressed to improve the beauty. However, this method has the possibility of chipping and layering of porcelain, which diminishes the good and acceptable results. In addition, the weak bond between porcelain and zirconia can make restoration less likely to succeed (Beuer et al. 2010). Full‐contour monolithic zirconia is used to overcome these problems. The most important advantages of monolithic zirconia restorations include an apparent reduction in the thickness of these types of restorations, as well as less tooth grinding, minimal wear of the anterior tooth, and long‐term clinical success (Beuer et al. 2012).

Translucency and color parameters strongly influence the adaptation of natural teeth to restorative materials. Translucency is inversely related to ceramic thickness. Unfortunately, reducing the thickness of ceramic materials reduces their strength (Heffernan et al. 2002); however, monolithic zirconia shows good mechanical properties even at minimal thicknesses (Zesewitz et al. 2014). Research has shown that the fracture toughness of monolithic zirconia crowns at lower thicknesses is also acceptable due to the very high flexural strength of zirconia (> 1000 MPa), so the authors concluded that the fracture resistance of YZP crowns is 1 mm thick equal to the metal‐ceramic coating. Therefore, various studies have reported the appropriate thickness of monolithic zirconia restorations to be 0.5–1 mm (Nakamura et al. 2015; Sun et al. 2014). As a result, translucent Y‐TZP may be used in the posterior region for crowns and FPDs instead of a tooth‐colored cast metal alloy. It may require minimal occlusal surface trimming with only 1 mm of occlusal thickness while providing both function and esthetics (Zesewitz et al. 2014). Recently, new compounds of Y‐TZP with different mechanical and visual properties have been offered for dental CAD‐CAM systems for short‐term monolithic restorations and conservative tooth preparation (Huettig and Gehrke 2016). High‐translucent zirconia has been proposed as an alternative to lithium disilicate for monolithic restorations due to its increased translucency and good mechanical properties, so it provides more beauty (Huettig and Gehrke 2016). The final color of a translucent material can be affected by the surface preparation, the color of the underlying tooth, the color of the cement, and the thickness of the restoration material. However, the possibility of achieving the desired color is reduced due to higher translucency for a patient with discolored teeth (Chaiyabutr et al. 2011). Considering the lack of previous studies about the ability to cover the color of discolored teeth by monolithic zirconia with high translucency, considering the factors affecting it, this study aims to assess the effect of the thickness of high‐translucency zirconia and the color of the substrate on the final color of the restoration. We always have the challenge of predictable esthetics with high‐translucent zirconia, according to its thickness and substrate shade's impact, and this study is conducted to overcome the problem.

## 2 Materials and Methods

The present study is a vitro research. A total of 60 monolithic zirconia disks with high translucency with a 10‐mm diameter were equally fabricated in two thicknesses of 6.0 and 1 mm on composite substrates with colors A2, A3/5, and C3. Considering a significance level of 5% and a test power of 80%, and according to the results of similar studies with an *S* = 1 (standard deviation [∆*E*] and to achieve a significant difference of at least 23.1 in the average ∆*E* in three groups, 10 repetitions in each group and 60 samples are needed due to two thicknesses and three colors on the substrate.

In this study, 60 monolithic zirconia ceramic disks with high translucency (Ceramil Zolid FX, Amann Girrbach, Germany) with a diameter of 10 mm and thicknesses of 7.0 and 1.1 mm with a special design were prepared using copy milling technique by computer‐aided device design and computer (CAD‐CAM) (Amann Girrbach, Germany) and exocad software. On one side of the disk, there was a circular hole with a depth of 0.1 mm and a diameter of 8 mm to create a uniform space between the disks and the composite substrates. As a result, on one side of each disk, a protrusion with a height of 0.1 mm was created, whereas the final thicknesses of the disks were 0.6 mm and 1 mm in the center. Then, the disks are dipped in colored liquid (Ceramil Liquid, Amann A2 Girbach, Germany) according to the instruction guide of the factory. After that, the samples were sintered in the temperature range of 8°C–1450°C per minute for 2 h. The size of the disks was measured with a digital micrometer (293 MDC‐MX Lite, Mitutoyo Corporation, Tokyo, Japan) with an accuracy of ±0.002 mm after sintering to ensure the accuracy of the thickness and size of the samples.

The disks were adjusted to achieve the desired thickness with a range of ±0.02 mm using a polishing kit (BruxZir; Glidewell Direct, Irvine, CA). For this purpose, the polishing kit (BruxZir; Glidewell Direct, Irvine, CA) was used, and the disks that did not have the desired thickness were excluded from the study. Also, disks with cracks and visible defects were excluded from the study and replaced with new samples with desirable characteristics. Then, all the samples were cleaned and dried for 15 min in an ultrasonic device containing 98% ethanol (Malkondu et al. 2016).

Resin composite disks in colors A3.5 and C3 were used to simulate the color substrate, as well as color composite (Estelite E Quick, Japan) A2 for the control group. A total of 20 samples in each composite color (A2, A3.5, and C3) were prepared. Then, the samples were polished using Grit 800 grit silicon carbide papers for 10 min and finally cleaned in an ultrasonic bath containing 98% ethanol for 15 min (Tabatabaian et al. 2016; Chen et al. 2015). Zirconia disks with a thickness of 6.0 mm (30 pieces in total) were randomly divided into three groups of 10 pieces. Zirconia disks with a thickness of 1 mm (30 pieces in total) were randomly divided into three groups of 10 pieces. A group of 10 of both 0.6 and 1 mm thicknesses was randomly selected on each color in the substrate, and thus six groups were formed:

– Group 1: 10 disks with 0.6 mm thickness and A2 substrate color.
– Group 2: 10 disks with 1 mm thickness and A2 substrate color.
– Group 3: 10 disks with 0.6 mm thickness and 3.5A substrate color.
– Group 4: 10 disks with 1 mm thickness and 3.5A substrate color.
– Group 5: 10 disks with 0.6 mm thickness and C3 substrate color.
– Group 6: 10 disks with 1 mm thickness and C3 substrate color.

The colorimetry of each of the samples was investigated twice: the first time by a drop of distilled water and the second time by cement on the composite infrastructure. The cement used in this study was resin cement (Panavia F2, Kurary, Tokyo, Japan), which is a self‐etch and dual‐cure cement.

In this study, the parameters of the samples were recorded based on the absorption intensity by a device (Vita easy shade, Zahnfabrik, USA). The aforementioned device can quantitatively report CIE *L*.*a*.*b* color standard parameters.

A clinical device called a spectrophotometer (Vita easy shade, Zahnfabrik, USA) was used to determine the color indices (*L*, *a*, and b) in the color system (CIELAB) of the samples. The spectrophotometer was calibrated according to the manufacturer's recommendations. The standard light situation was under D65 light. The location was CIElab. A small amount of silicone putty (Speedex; Coltene, Altstatten, Switzerland) was molded around the tip of the device and the substrate disc assembly to make a mold. This mold made the spectrophotometric position of all the samples uniform, prevented the passage of external light, and stabilized the position of the samples, substrates, and devices.

The measurements were repeated three times for each sample, and the average of three numbers in each color index was recorded as the final number of that index (Malkondu et al. 2016). Then the samples were completely dried and cemented with PANAVIA SA Cement according to the manufacturer's instructions on the corresponding substrate, and the process of determining the color indices was repeated. Finally, ∆*E* was calculated according to the following equation:

(1) $$\Delta E_{\text{ab}}^{*}={\left[{\left(L_{2}^{*}-L_{1}^{*}\right)}^{2}+{\left(a_{2}^{*}-a_{1}^{*}\right)}^{2}+{\left(b_{2}^{*}-b_{1}^{*}\right)}^{2}\right]}^{1/2}$$

where *L* represents the luminosity level in a material (between 0 [black] and 100 [white]), *a* represents green to red, and *b* represents blue to yellow. A lower ∆*E* indicates a greater color similarity between the two compared objects, and different ∆*E*s are used to differentiate colors due to the variation in the capacity of the human eye to distinguish between colors.

In this study, the clinically acceptable threshold of ∆*E* was considered < 5.5, and the ideal threshold of ∆*E* (clinically unintelligible) was considered < 2.6 (Oh and Kim 2015; Douglas et al. 2007; Douglas and Brewer 1998). Repeated measures ANOVA procedure was used for statistical analysis of the data, and a one‐sample *t*‐test was used in SPSS 20 to compare ∆*E* values, for acceptability and understandability, respectively, with threshold values of 5.5 and 2.6. This study has been approved by the Research Ethics Committee of Shahid Sadoughi University of Medical Sciences, Yazd with ID IR.SSU.REC.1396.138.

## 3 Results

The data distribution was analyzed using Kolmogorov–Smirnov and Shapiro–Wilk tests, which showed that the data were normal in all three indices *L*, *a*, and *b* (Tables 1, 2, 3).

**Table 1:** The mean and standard deviation of the *L* color index in the examined samples.

Intermediate	Substrate color	Disc thickness	Mean	Standard deviation
No cement (water)	A2	0.6	75.58	1.916
		1	77.82	1.69
	A3.5	0.6	73.23	0.78
		1	78.15	2.01
	C3	0.6	71.00	0.82
		1	74.72	1.00
Cement	A2	0.6	76.95	1.17
		1	76.03	0.82
	A3.5	0.6	75.88	1.93
		1	76.65	1.84
	C3	0.6	73.46	2.20
		1	74.12	1.18

**Table 2:** The mean and standard deviation of color index *a* in the studied samples.

Intermediate	Substrate color	Disc thickness	Mean	Standard deviation
No cement (water)	A2	0.6	0.220	0.325
		1	0.590	0.747
	A3.5	0.6	0.640	0.254
		1	1.150	0.805
	C3	0.6	0.15	0.217
		1	0.710	0.600
Cement	A2	0.6	1.190	2.752
		1	1.090	0.438
	A3.5	0.6	1.31	2.72
		1	1.37	0.44
	C3	0.6	0.200	0.194
		1	0.92	0.470

**Table 3:** The mean and standard deviation of color index *b* in the studied samples.

Intermediate	Substrate color	Disc thickness	Mean	Standard deviation
No cement (water)	A2	0.6	8.73	0.86
		1	8.28	3.13
	A3.5	0.6	9.94	0.58
		1	9.64	2.43
	C3	0.6	8.68	0.75
		1	9.19	2.05
Cement	A2	0.6	9.91	0.93
		1	10.73	1.56
	A3.5	0.6	10.48	1.14
		1	11.51	1.71
	C3	0.6	9.95	0.88
		1	10.28	1.82

### 3.1 Thickness

**Figure 1:**
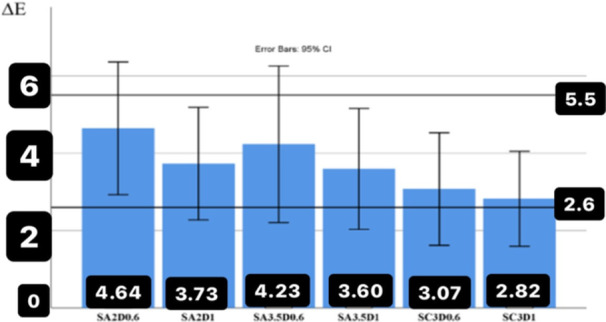
The average values of color difference (∆*E*) between zirconia disks with two thicknesses of 0.6 and 1 mm (substrates with the same color and without the presence of cement).

The average results of ∆*E* values between 0.6 and 1‐mm thick zirconia disks on the substrate with the same color according to the color of the substrate are presented in Figure 1 to investigate the effect of thickness on the color difference (∆*E*). In addition, the lower numbers obtained with fixed numbers of 5.5 and 2.6 were analyzed by the one‐tailed *t*‐test to investigate the effect of thickness on ∆*E* values. The maximum effect of the thickness difference on ∆*E* was observed on the color of the A3.5 substrate with a color difference of ∆*E* = 5.65 ± 2.27 in the absence of cement and with the intermediate material of water, which is significantly higher than 5.5. (*p* = 0.244). In addition, the minimum effect of thickness on the substrate color of the C3 was observed with a color difference of ∆*E *= 4.15 ± 1.25, which is significantly < 5.5 (*p *= 0.004 (Figure 1).

### 3.2 Substrate Color

**Figure 2:**
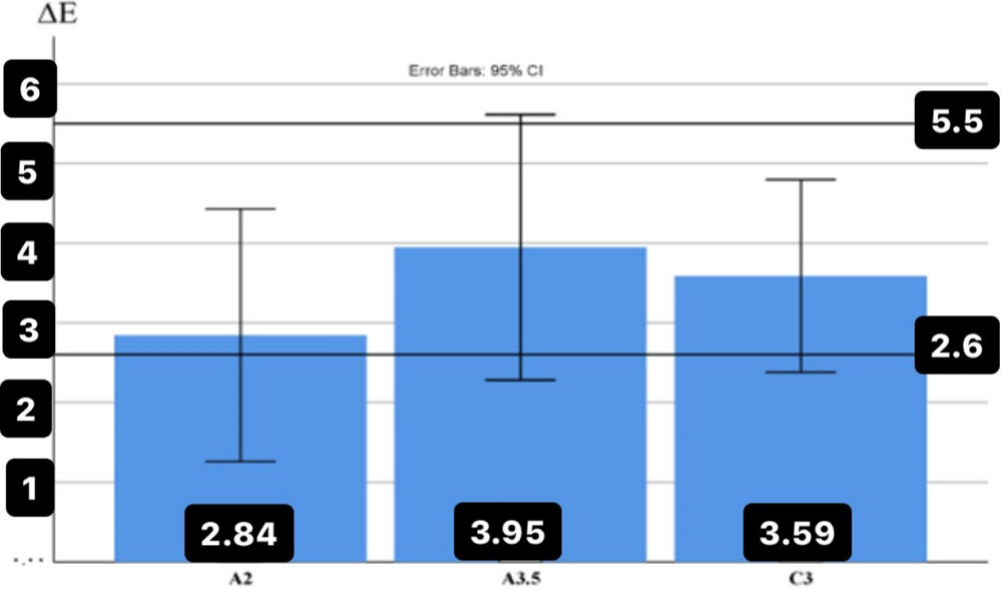
The average values of the color difference (∆*E*) between zirconia disks with two thicknesses of 0.6 and 1 mm (substrates with the same color and with the presence of cement).

The average results of ∆*E* values between 0.6 and 1‐mm thick zirconia disks on the substrate with the same color according to the color of the substrate are presented in Figure 2 to investigate the effect of thickness on the color difference (∆*E*).

In addition, the lower numbers obtained with fixed numbers of 5.5 and 2.6 were analyzed by the one‐tailed one‐sample *t*‐test to investigate the effect of thickness on ∆*E* values. The maximum effect of the thickness difference on ∆*E* was observed on the color of A3.5 substrate with cement (∆*E* = 3.95 ± 2.32) with a value of ∆*E* significantly lower than the value of 5.5 (*p* = 0.032), and the minimum effect of thickness on substrate color of ‌A2 was observed (∆*E *= 2.84 ± 2.21), which is significantly < 5.5 (*p* = 0.004) (Figure 2).

The restoration of the average color difference of zirconia disks with the same thickness on sub‐structures of colors A3/5 and C3 compared to the control group (with the A2 sub‐structure color) was obtained to analyze the effect of sub‐structure color on the final color. This comparison was performed without the presence of cement (with water as an intermediate material), and its results were shown separately by the thickness (0.6 and 1 mm) and substrate color (5.3A and C3).

**Figure 3:**
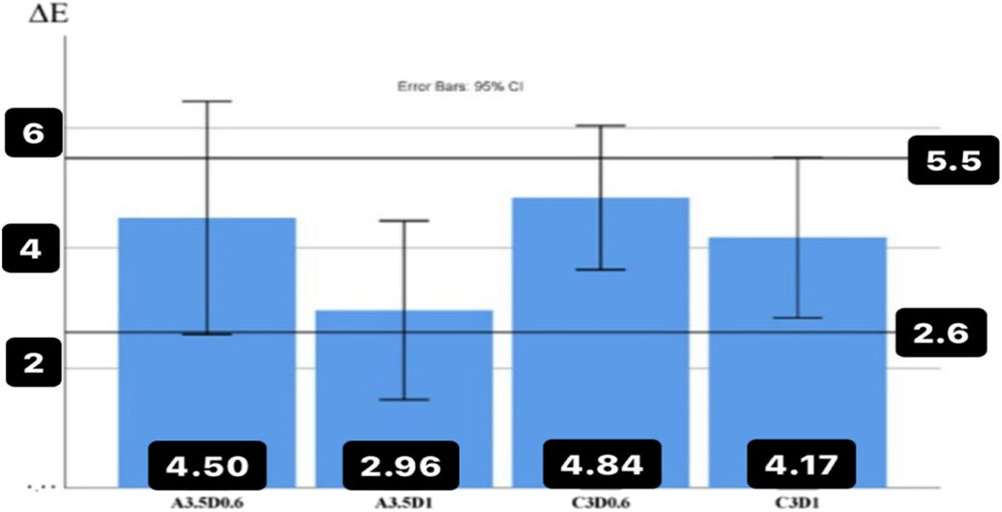
The average values of the color difference (∆*E*) between different substrates compared to the control group (with the same thickness and without cement).

The maximum color difference with the control group was observed in the colors of substrate C3 and thickness of 0.6 mm (∆*E* = 4.84 ± 1.67, which lower value was with a fixed number of 5.5 by one‐sample *t*‐test. It showed no significant difference (*p* = 0.121), which means that this value is in the upper limit of 5.5 values and the range of unacceptable color difference of the study. The minimum color difference with the control group was observed in the A3.5 substrate color with a thickness of 1 mm (∆*E* = 2.961 ± 2.08), which was significantly < 5.5 (*p* = 0.002) and was within the acceptable range of the study (Figure 3).

**Figure 4:**
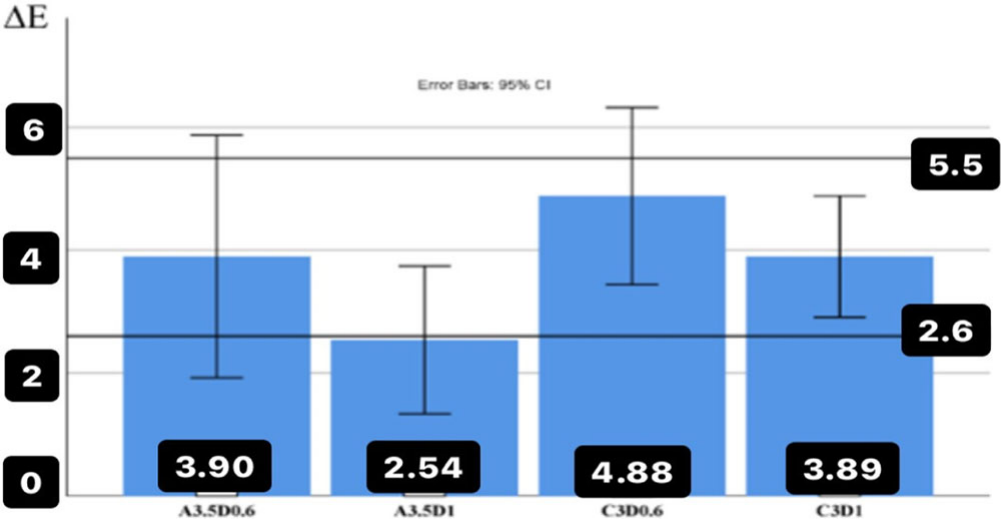
The average values of the color difference (∆*E*) between different substrates compared to the control group (with the same thickness and with the intermediate material of cement).

The restoration of the average color difference of zirconia disks of the same thickness was obtained on substrates colored A3/5 and C3 with the control group having a substrate color of A2. This comparison was performed with cement to analyze the effect of substrate color on the final color, which is shown separately by the thickness (0.6 and 1 mm) and the color under the structure (5.3A and C3) in Figure 4. The maximum color difference with the control group in the presence of cement was observed in the group with C3 substrate color and zirconia disc thickness of 0.6 mm (∆*E* = 4.88 ± 2.01). This difference (∆*E*) is in the unacceptable limit of the study (*p* = 0.178) compared to 5.5, and its lower value with a fixed number of 5.5 was checked by a one‐sample *t*‐test (indicating the significance of this lack of difference). This means that this value is in the upper limit of 5.5 values and the range of unacceptable color difference. The minimum color difference with the control group in the presence of cement was observed in the group with A3/5 substrate color with a thickness of 1 mm (∆*E* = 2.54 ± 1.67). This difference (∆*E*) is in the acceptable limit of the study (*p* = 0.000) when compared to 5.5; however, compared to 2.6, this color difference is not within the ideal limit of the study (*p *= 0.455). Therefore, it was significantly < 5.5 and was within the acceptable limit of the study.

The maximum and minimum color difference (∆*E*) was observed in the aforementioned groups without cement. In addition, it can be seen from graphs 3 and 4 that the values of ∆*E* decrease with the increase in thickness (Figure 4).

### 3.3 Presence/Absence of Cement

**Figure 5:**
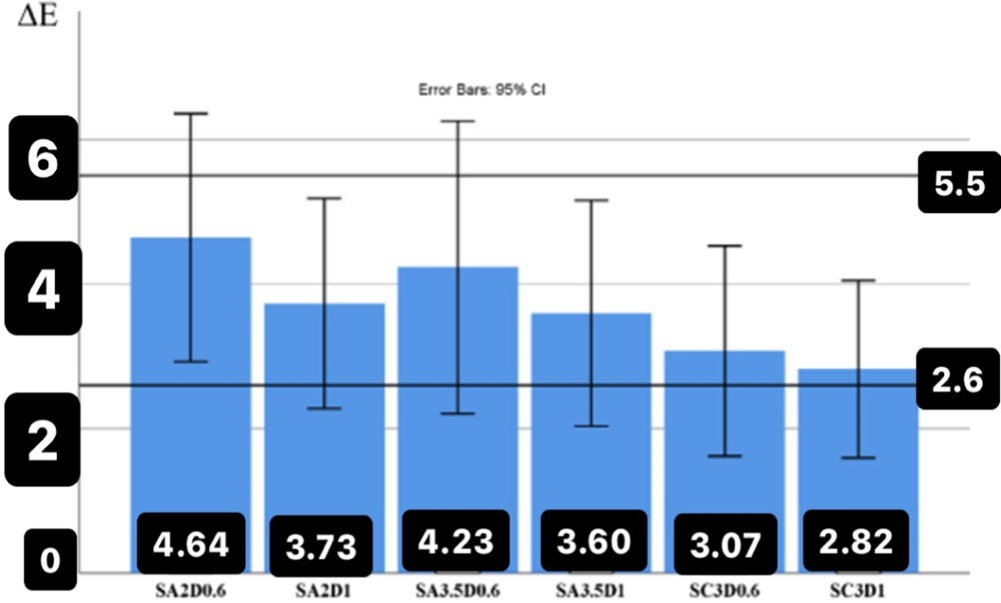
The average values of the color difference (∆*E*) in the presence of cement compared to water (same thickness and color of substrate).

The average color difference (∆*E*) of zirconia disks (with similar thickness and substrate color) was obtained in the presence and absence of cement to analyze the effect of cement on the color difference (Figure 5). The maximum effect of cement on ∆E values was observed on the color substrate A2 at a thickness of 0.6 mm (∆*E *= 4.64 ± 2.39, which was in the range higher than 5.5 and unacceptable (*p *= 0.144). The minimum effect of cement on the color difference (∆E) was observed on the substrate color of C3 with a thickness of 1 mm (∆*E *= 2.82 ± 1.71), which was significantly less compared to 5.5 (*p* = 0.001). The comparison of the color difference values (∆E) with a constant number of 5.5 showed that the presence of cement had the maximum effect on the 0.6 mm zirconia disks and on the substrates with the color difference (4.64 ± 2.2 A239) (∆*E *= 0.144 (*p *= ) and substrate color is A3.5 with the color difference (4.23 ± 2.83) (∆*E *= 0.095 (*p *= ) (Figure 5).

## 4 Discussion

Azer et al. reported that the thickness of the ceramic and the characteristics of the substrate can affect the color of the ceramic laminates. Although the translucency level of the laminates is different from that of zirconia, the results of this study are consistent with the results of the present study (Azer et al. 2011). Tabatabaian et al. (2019) investigated the effect of zirconia ceramic thickness on the paint coating ability. They reported that the ability to cover ceramic paint increases with the increase of ceramic thickness, which is in line with the results of the present study. They also found that a minimum thickness of 0.4 mm of zirconia disk is needed to achieve the ideal color coverage in the case of A1 and A3.5 composite substrates, but in our study, the minimum thickness of 0.6 mm (with the presence of cement) could cover the color of A3.5 substrates in an acceptable and not ideal range. This problem can be due to the different types of zirconia used in the two studies and the fact that the zirconia in our study is more translucent, which requires more thicknesses of zirconia and the help of cement to cover the color of substrates (Tabatabaian et al. 2019). Kumagai et al. reported a significant color difference between the thickness of 0.3 and 0.5 mm zirconia crowns on two different tooth colors. Although other thicknesses and other colors were examined in the present study, both studies showed the possibility of the substrate color affecting the final color when using coatings with smaller thicknesses (Kumagai et al. 2013).

Xing et al. investigated the effect of ceramic thickness and resin cement color on the color matching of ceramic veneers in discolored teeth. They found that the color change (∆*E*) of ceramic veneers is significantly different from the measured area of the tooth, the thickness of the ceramic, and the shade of the resin cement. They also concluded that the amount of ∆*E* in the thickness of 0.5 mm is more than that of 0.75 mm, which was inconsistent with the results of our study regarding the effect of ceramic thickness on *E* (Xing et al. 2017). Li et al. (2009) about the effect of the composite blind color showed that the composite color had a definite effect on the final color of all ceramics (Li et al. 2009). In addition, during a laboratory study, Amirpour et al. investigated the effect of four different types of infrastructure on the final color of polymer‐infiltrated ceramics (PICN). They found that the type of substrate restoration had a significant effect on the mean color difference (Δ*E*) values of restorations fabricated from VITA Enamic. The color difference in all four infrastructure restoration groups was above the detectable threshold in the clinic. Both of the mentioned studies are consistent with the results of the present study regarding the effect of substrate color on the final color of the restoration. However, Dorriz et al. found that the values of *L*, *a*, and *b* of zirconia restorations on three types of substrates were not significantly different, and the reason for the substrate's lack of influence on the final color was probably the lack of translucency of zirconia, which could not show the substrate color. This change of *E* in the 0.6 mm zirconia disk on the A2 substrate reflexes as an unacceptable color change because when our zirconia disk is A2 in color and the color of the substrate is also A2, it is desirable that color difference will not appear by cementing. Because the problem of color coverage is not discussed here, by analyzing the results, it was found that Panavia cement can increase ∆*E* in this case, higher than the acceptable limit (*p* = 0.144), which can be due to the coverage of Panavia cement and to some extent opacity that the zirconia disc with high translucency in low thickness reflected the color of its substrate cement and made this unfavorable ∆E. Of course, it was found that by increasing the thickness of the disc to 1 mm, this ∆*E* is within the acceptable limit of the study, which can be due to the greater thickness of the disk, which prevents the effect of the color of the cement on the final color of the disk and decreases its effect on the values of ∆*E*.

The effect of cement on the ∆*E* values in 0.6 mm zirconia on the A3/5 substrate, as well as on the A2 substrate, is high. Therefore, it has increased the ∆*E* to higher than the 5.5 values, which indicates the change of ∆*E* in the case of the color of the substrate. The A3/5 is considered an advantage because the more ∆*E* the cement can create compared to the state without cement which means its capability to cover more of the substrate structure. Of course, this effect is reduced by increasing the thickness of the disk to 1 mm because the greater thickness prevents the cement from being seen from behind the disk and creates noticeable effects of the cement on ∆*E*. So, according to the color of the A3/5 substrate, it concludes that we can achieve a favorable color coverage with the presence of Panavia cement with the use of the minimum thickness of the zirconia disk (0.6 mm), and there is definitely no need to increase the thickness of the disk and, as a result, grind more teeth to create the desired final color of the restoration.

The results of the study showed that the presence of cement in the C3 color substrate, even in the low thickness of the zirconia disk, cannot have a noticeable effect on the ∆*E* values, to the extent that it can have a covering effect for the substrate. This can be due to the more dominant effect of the color of the C3 substrate, which shows that we cannot be satisfied with the effect of cement alone to cover its color. In addition, we must increase the thickness of the disc to create the desired final color. The results of our study were consistent with the study of Chang et al. They investigated the effect of different resin cements on the color of different all‐ceramic veneers and concluded that resin cements significantly affected the final color of these ceramics (Chang et al. 2009). Malkondu et al. investigated the effect of cement type on the color and translucency of translucent monolithic zirconia. They reported that the color and translucency of zirconia disks changed significantly after cementation. Reducing the thickness of zirconia from 1 mm to 0.6 mm significantly increases translucency, and the effect of cement on color also increases, which was also observed in the present study. Malkondu et al. found that the final color and translucency of zirconia samples were affected by translucency and cement color. They reported that the cement used in this study did not cause unacceptable color changes, with the exception of resin cement, which had unacceptable changes for the thickness of 0.6 mm. In the present study, the use of Panavia cement for a thickness of 0.6 mm on the A2 substrate caused unacceptable color changes (Malkondu et al. 2016). Tabatabaian et al. (2016) investigated the effect of cement on the characteristics of zirconia ceramic color in a laboratory study. They reported that Glass Ionomer and Panavia F2.0 cements produce acceptable color changes. The results of the present study in relation to creating acceptable color changes when using Panavia cement on the substrate with 3.5 colors are consistent with Tabatabaiyan et al. (Kim et al. 2013).

Pires et al. analyzed the effect of ceramic type, substrate, and cement on the visual color of lithium disilicate ceramic (IPS e.max Press). The results of their study were as follows: the color of the substrate, the type and thickness of the ceramic, and cement significantly affect the visual characteristics of the color, and in the present study, substrate color and cement could affect the final color (Pires et al. 2017). Begum et al. investigated the effect of ceramic thickness and shade of cement on the ability to cover the color of laminate veneers and reported that the ability to cover the color of ceramics used for laminate veneer is significantly affected by the thickness and shade of cement, which is also found in the present study. The relationship between thickness and ∆*E* and the effect of thickness on paint coverage was observed in such a way that ∆*E* was higher in 0.6 mm thickness compared to 1 mm when different substrate colors were used. In addition, the use of cement could also affect the final color (Begum et al. 2014). According to the results of the present study, the first null hypothesis about the lack of difference between two thicknesses of monolithic zirconia with high translucency in creating different ∆*E* values was rejected. Moreover, the second hypothesis of the study about the acceptability of ∆*E* values in two groups of zirconia with the same thickness but with substrates having different colors (C3 and A3.5) was relatively rejected compared to the control group (A2). That is, the values of ∆*E* in the thickness of 1 mm of zirconia on the A3.5 and C3 substrates (with and without the presence of cement) and the thickness of 0.6 mm of zirconia on the A3.5 substrates with the presence of cement were clinically acceptable (∆*E* < 5.5 and also the third hypothesis about the lack of effect of cement on ∆*E* values was rejected.

## 5 Conclusion

Changing the thickness of zirconia ceramic can affect ∆*E*. Increasing the thickness of zirconia ceramic can reduce ∆*E* values and thus increase the ability to cover the color of the substrate. It is possible to acceptably cover the color of the A3.5 substrate in the presence of Panavia cement using a minimum thickness (0.6 mm) of high‐translucency zirconia ceramic. A minimum thickness of 1 mm zirconia disc with high translucency (with and without cement) is needed for the substrate coating in 3C color. The use of Panavia cement in small thicknesses of zirconia disks (0.6 mm) on the substrates of 2A and 3.5A can have a significant effect on ∆*E*. The use of Panavia cement for cementing zirconia disks with high translucency in color A2 and thickness of 0.6 mm on the substrate in color 2A increases the values of ∆*E* and causes unacceptable color changes. The presence of Panavia cement does not have a significant effect on the ∆*E* values despite the presence of C3 substrate color, and to cover the 3C substrate color, we must pay attention to the problem of more tooth grinding and increasing the thickness of the translucent zirconia disc. In this study, a brand of zirconia with high translucency was used. Future studies should compare different brands of high‐translucency zirconia.

## Author Contributions

**Mahnaz Hatami:** conceptualization, methodology, investigation, writing–original draft. **Elham Jalali:** data curation, writing–review and editing. **Mohamad Hossein Lotfi Kamran:** resources, visualization. **Alireza Danesh Kazemi:** formal analysis, software. **Amirhossein Fathi:** project administration, writing–review and editing.

## Conflicts of Interest

The authors declare no conflicts of interest.

## Data Availability

Data will be available on request due to privacy restrictions.
